# The Occurrence Characteristics and Ecological Risks of Antibiotics in Surface Water and Groundwater of the Huixian Wetland

**DOI:** 10.3390/toxics13060460

**Published:** 2025-05-30

**Authors:** Dunqiu Wang, Min Li, Chenchen Liao, Kun Dong, Yi Yao

**Affiliations:** 1 Guangxi Key Laboratory of Environmental Pollution Control Theory and Technology, Guilin University of Technology, Guilin 541004, China; wangdunqiu@glut.edu.cn (D.W.); limin_am@163.com (M.L.); 18060127792@163.com (C.L.); 2 Engineering Research Center of Watershed Protection and Green Development, Guilin University of Technology, Guilin 541006, China; 3 Guangxi Engineering Research Center of Comprehensive Treatment for Agricultural Non-Point Source Pollution, Guilin University of Technology, Guilin 541006, China; 4 Modern Industry College of Ecology and Environmental Protection, Guilin University of Technology, Guilin 541006, China; 5 Collaborative Innovation Center for Water Pollution Control and Water Safety in Karst Area, Guilin University of Technology, Guilin 541006, China

**Keywords:** antibiotic, Huixian Wetland, ecological risk, health risk

## Abstract

The concentrations of 17 antibiotics in the surface water and groundwater of a typical river in the Huixian Wetland were measured, and the ecological and health risks of these antibiotics to surface water and groundwater were assessed. The three types of antibiotics measured included quinolones, tetracyclines, and sulphonamides. The results showed that the mean values of the three antibiotics in surface water were sulfonamides > quinolones > tetracyclines and in groundwater were sulfonamides > tetracyclines > quinolones, and the antibiotic residues were associated with aquaculture and livestock breeding in the wetland, which indicated that antibiotics had a very high rate of use in the Huixian Wetland. The results of the ecological risk evaluation showed that the potential risk of five antibiotics, namely ofloxacin, sulfadiazine, sulfamethoxazole, enrofloxacin, and doxycycline, was high. The results of the health risk evaluation indicated that most of the residual antibiotics were of a medium and low risk to humans.

## 1. Introduction

Antibiotics are classified into three categories based on their origin: natural compounds derived from microorganisms, semi-synthetic agents created by chemically modifying natural substances, and fully synthetic compounds. They are widely used in medicine and aquaculture to prevent or treat human and animal diseases [1]. In agricultural, livestock, and aquaculture industries, antibiotics are employed as feed additives to promote animal growth. However, the emergence and development of antibiotic resistance in the environment, coupled with excessive antibiotic use, pose severe threats to public health systems, aquatic ecosystems, and human health. These environmental pollution issues have garnered global attention [2]. Over the past decades, hundreds of antibiotics have been extensively utilized for disease prevention and treatment in humans and animals, as well as in animal feed to enhance growth and fattening efficiency. In 2020, global veterinary antibiotic consumption reached 99,502 tons, and it is projected to increase by 8% to 107,472 tons by 2030 [3]. China has consistently ranked among the world’s top producers and consumers of antibiotics [3], accounting for approximately 20% of global usage, with veterinary antibiotics constituting 52% and human antibiotics 48% [4]. Annually, around 24,700 tons of antibiotics are discharged into water bodies. Over 90% of consumed antibiotics enter the environment as parent compounds or metabolites via feces or urine after biological excretion. After administration, 5–90% of antibiotics may be excreted as metabolites or parent compounds and enter aquatic ecosystems predominantly through wastewater effluents and aquaculture, posing dual environmental risks as priority contaminants due to their chronic toxicity to non-target organisms and persistent induction of antimicrobial resistance via selection pressure, while their continuous discharge reinforces their classification as “pseudo-persistent” pollutants [5]. Urban wastewater treatment plants, hospitals, and livestock farms are the primary sources of antibiotics in surface water [6]. Although antibiotics are considered metastable pollutants, even at low concentrations, they can induce the proliferation of antibiotic-resistant bacteria (ARB) and antibiotic resistance genes (ARGs), posing significant ecological and human health risks [7]. Antibiotics not only contribute to organic chemical pollution but also promote ARB and ARG formation, threatening human health and ecosystems through food chains [8].

Sulfonamide antibiotics are widely used synthetic antibiotics in disease treatment, fisheries, and aquaculture due to their broad antibacterial spectrum, stability, and efficacy [9,10]. Fluoroquinolones have the characteristics of strong antibacterial activity, stable metabolism, low production costs, and no cross-resistance with other antibacterial drugs. They are widely used in the world [11]. Tetracyclines, similar to the aforementioned classes, are broad-spectrum bacteriostatic antibiotics stable in acidic media and resistant to environmental oxidation, forming low-volatility and low-degradation byproducts [12]. Residues of sulfonamides, fluoroquinolones, and tetracyclines have been detected in diverse environmental matrices, including rivers [13], wastewater treatment plants [14,15,16], soils [17], and sediments [18,19,20]. China’s annual antibiotic consumption is approximately 162,000 tons, with a daily defined dose (DID) per 1000 inhabitants nearly six times higher than in Europe and the United States [21]. Contamination by sulfonamides, fluoroquinolones, and tetracyclines has been confirmed in multiple Chinese water systems, including Honghu Lake and surrounding waters in Hubei [22], Nanming Lake in Guiyang [23], the Yangtze River Basin [24], Yellow River Basin [25], Haihe River Basin [26], Huangpu River Basin [27], Liao River Basin [28], and rivers in Hong Kong’s Yuen Long, Kam Tin, and Shing Mun regions [29]. Internationally, sulfamethoxazole, flumequine, oxolinic acid, and nalidixic acid residues (15 μg/kg, 32 μg/kg, 16 μg/kg, and 19 μg/kg, respectively) were observed in Seine River floodplain sediments, while 17 antibiotics (ND–544 ng/L) were detected in Seine River water [30]. High antibiotic concentrations (e.g., oxytetracycline: 1100 ng/L; trimethoprim: 1700 ng/L; sulfamethoxazole: 2400 ng/L) were reported in Lahore’s rivers, Pakistan [31].

With rapid population growth and socioeconomic development in China, water pollution in karst wetland ecosystems has intensified. The Huixian Karst Wetland, a unique ecosystem integrating wetland and karst features, plays critical roles in water purification, runoff regulation, and groundwater recharge. However, non-point source pollution from intensive agriculture and aquaculture has exacerbated water quality degradation, weakening wetland functions and exposing precious karst groundwater resources to contamination risks [32]. Karst aquifers, characterized by calcium-rich alkaline water and rapid pollutant infiltration through sinkholes, exhibit dynamic seasonal variations in water levels and flow rates. Previous studies on antibiotic residues in Huixian Wetland focused on surface water, groundwater, and sediment distribution in core protected areas, yet no data exist on the trace-level contamination of sulfonamides, tetracyclines, and fluoroquinolones in the ancient Guiliu Canal, Mudong River, and Huixian River systems. This study addresses this gap by investigating the spatial distribution, sources, and ecological risks of these antibiotics in surface and groundwater. Building on prior research [32], our work not only quantifies antibiotic concentrations but also identifies pollution sources via principal component analysis (PCA), offering insights for pollution management. This study builds on prior investigations of antibiotic contamination in the Huixian karst wetland by expanding the scope from sulfonamides (SAs) to include quinolones (QNs) and tetracyclines (TCs), while refining source attribution and risk assessment methodologies. Compared to earlier work focused exclusively on SAs, the current findings reveal broader contamination patterns and nuanced ecological-health linkages, aligning with regional trends while highlighting the unique vulnerabilities of karst aquatic systems.

The objective of the study is (1) investigate the spatiotemporal distribution of sulfonamides, fluoroquinolones, and tetracyclines in surface and groundwater of the Huixian Karst Wetland and (2) assess the ecological and human health risks posed by individual and mixed antibiotics using risk quotient (RQ) and mixed hazard quotient (MHQ) methodologies. Antibiotics were quantified via ultra-performance liquid chromatography-tandem mass spectrometry (UPLC-MS/MS). Ecological risks were evaluated using RQ and MHQ, while health risks were assessed via ingestion pathway risk quotients (RQ_H_).

## 2. Materials and Methods

### 2.1. Sample Collection

**Figure 1:**
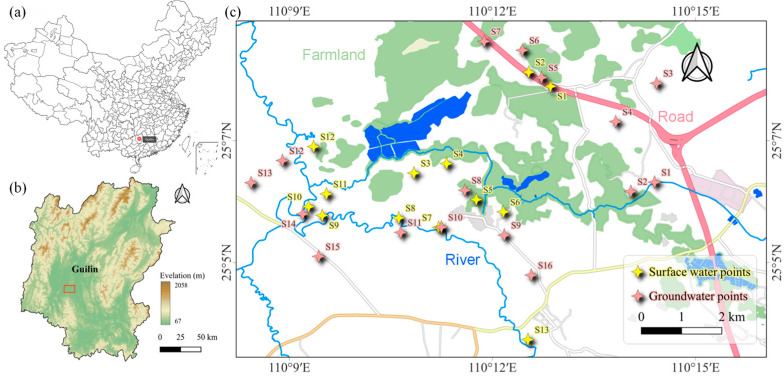
(**a**) Location of Guilin. (**b**) Location of sampling area in Guilin. (**c**) Locations of water sampling points.

The Huixian Karst Wetland (110°08′15″–110°18′00″ E, 25°01′30″–25°11′15″ N), located in Huixian Town, Lingui District, Guilin City, Guangxi, is one of the largest low-altitude karst swamp wetlands in China. It has an elevation of 150–160 m and a total area of 120 square kilometers [33]. The study area is the core zone of the Huixian Wetland, with the main study sections selected along the ancient Gui-Liu Canal, Mudong River, Huixian River, and Xiangsi River. Based on the hydrological characteristics of the Huixian Wetland and its surrounding inflow systems, this study quantified the pollution levels of antibiotic pharmaceuticals in the Huixian Wetland system by sampling surface water and groundwater in key areas. A total of 29 sampling sites were selected (Figure 1). Among these, 16 groundwater samples (collected at depths of 10–20 m) were obtained from wells directly used as drinking water sources by local residents. These wells are located in villages with frequent human activity, including Fengjia Village, Mian Village, Huangchatang Village, Wencun Village, Gaoshan Village, and Mojia Village. Surface water sampling sites 3–6 were along the ancient Gui-Liu Canal, sites 7–9 and 13 were along the Huixian River, and sites 10–12 were along the Luoqing River. Surface water sites 3–4 are near aquaculture zones, and sites 10–12 are near livestock farming zones, while the remaining surface water sites are adjacent to agricultural cultivation zones. Sampling was conducted in December 2024. All water samples were collected using a cylindrical sampler at approximately 0.5 m below the surface and stored in clean glass bottles (2.5 L). All samples were immediately placed in an ice environment and transported to the laboratory as soon as possible. Surface water samples were collected in brown borosilicate glass bottles at each sampling site. The bottles were pre-washed with methanol and deionized water and then rinsed twice with water from the sampling site before collection. Water samples were maintained at 4 °C, and the target antibiotics were extracted from the samples within two days. Field blanks were prepared by collecting ultrapure water in the same volume as the real samples. The same sampling containers as those used for real water samples were utilized, and they were pre-cleaned and sterilized. Preservatives were added at the same proportion as in the real samples. At the sampling site, the blank sample containers were opened and exposed to the ambient environment. Subsequently, the ultrapure water containing preservatives was poured into the containers. After sealing, the field blanks were transported and stored alongside the real samples.

### 2.2. Instruments and Reagents

The major instruments used in this study were the Waters Xevo TQ-S Micro ultra-performance liquid chromatography triple quadrupole mass spectrometer, Waters Oasis HLB solid-phase extraction columns (500 mg, 6 mL, Waters Corporation, Milford, MA, USA), nitrogen evaporator (N-EVAP-112, Organomation, Berlin, NH, USA), solid-phase extraction manifold (24-port model, Supelco, USA), ACQUITY UPLC BEH C18 chromatographic column (2.1 mm × 100 mm, 1.7 μm), Agilent Bond Elut SAX strong anion-exchange column (500 mg, 6 mL, Agilent Technologies, Santa Clara, CA, USA), mixed cellulose ester membrane filters (0.45 μm, 47 mm, Millipore (MilliporeSigma), Billerica, MA, USA), hydrophobic polytetrafluoroethylene (PTFE) syringe filters, and 1 mL disposable syringes. The target compounds were 17 antibiotics, including the sulfonamides (SAs) sulfadiazine (SDZ, CAS: 68-35-9), sulfamethoxazole (SMZ, CAS: 723-46-6), sulfamerazine (SMR, CAS: 127-79-7), sulfamethazine (SMM, CAS: 57-68-1), sulfapyridine (SMP, CAS: 80-35-3), sulfachloropyridazine (SCP, CAS: 80-32-0), sulfadoxine (SDM, CAS: 2447-57-6), sulfamonomethoxine (SM2, CAS: 1220-83-3), and trimethoprim (TMP, CAS: 738-70-5); the quinolones norfloxacin (NOR, CAS: 70458-96-7), ciprofloxacin (CIP, CAS: 85721-33-1), enrofloxacin (ENR, CAS: 93106-60-6), and ofloxacin (OFL, CAS: 82419-36-1); and the tetracyclines tetracycline (TC, CAS: 60-54-8), doxycycline (DC, CAS: 564-25-0), oxytetracycline (OTC, CAS: 79-57-2), and chlortetracycline (CTC, CAS: 57-62-5).

### 2.3. Impact Assessment Method of PTE Pollution in Soil

Surface water samples were filtered through glass fiber filter membranes (GF/F, 0.45 μm). The 17 selected antibiotics were extracted from the water samples using a 24-position solid-phase extraction (SPE) apparatus (Supelco, Merck, Bellefonte, PA, USA) with hydrophilic–lipophilic balance (HLB) sorbent columns (6 mL, 500 mg, Waters Corporation, Milford, MA, USA). Ultrasound-assisted extraction was used to process the precipitates, followed by purification using SAX columns coupled with HLB columns. Methanol was added to the water samples at a methanol-to-water volume ratio of 1:200 to inhibit microbial growth and facilitate antibiotic preservation. Surface water samples (1000 mL) were filtered through glass fiber filter membranes (GF/F, 0.45 μm) using a vacuum pump to remove visible particles, and 500 mL of the filtered water sample was measured into a beaker. Prior to extraction with Oasis HLB columns, 0.5 g of Na_2_EDTA and 50 ng of surrogate standards (sulfathiazole-D4, norfloxacin-D5, and atrazine-C3) were added to 1000 mL of a water sample. The sample was stirred with a glass rod, and the pH was adjusted to 3 using 1 mol/L hydrochloric acid. The HLB column was preconditioned sequentially with 6 mL of methanol, 6 mL of ultrapure water (pH = 3), and 6 mL of 2 g/L Na_2_EDTA at a flow rate of 3 mL/min. Upon completion of the injection of the 500 ml sample in the beaker, the HLB column was rinsed with ultrapure water (pH = 3) and vacuum-dried for 240 min. Finally, the analytes were eluted using 4 mL of methanol containing 0.1% (*v*/*v*) formic acid and 4 mL of methanol containing 3% (*v*/*v*) ammonia. The eluates were concentrated to near dryness using a nitrogen stream in a 50 °C water bath and reconstituted to 1 mL with a mixed solution of 0.05% (*v*/*v*) formic acid in water–methanol (3:2, *v*/*v*). The sample was then filtered through a 0.22 μm membrane into a brown sample vial for further analysis.

Analysis was performed using a Waters Xevo TQ-S Micro ultra-performance liquid chromatography coupled with a triple quadrupole mass spectrometer. The chromatographic conditions were set as follows: column temperature at 30 °C, injection volume of 5 μL, and mobile phases consisting of ultrapure water with 0.05% (*v*/*v*) formic acid (A) and methanol (B). The flow rate was maintained at 0.25 mL/min. The gradient elution program was as follows: at 2 min, the ratio of mobile phase A to B changed from 95/5 to 80/20; at 5 min, the ratio changed to 65/35; at 7 min, the ratio changed to 5/95; and at 9.5 min, the ratio returned to 95/5, with elution concluding at 12 min. The mass spectrometer was operated in positive electrospray ionization (ESI) mode and multiple reaction monitoring (MRM) mode [32] (Table 1). The ion source temperature was set to 150 °C, and the capillary voltage was set to 4000 V.

### 2.4. Sample Pretreatment

The laboratory quality assurance and quality control elements included laboratory blanks and field blanks. No analytes of interest were detected in the blank samples. The retention times, linear equations, correlation coefficients (R^2^), limits of detection (LODs), and limits of quantification (LOQs) for the 17 antibiotics are presented in Table 2. The LOD ranged from 0.01 to 1.02 ng·L^−1^, and the LOQ ranged from 0.04 to 3.25 ng·L^−1^. The recovery rates for spiked samples at a concentration of 5 ng·L^−1^ were between 76.64% and 110.65%. The limits of detection (LODs) and quantification (LOQs) in Table 2 were determined using the signal-to-noise ratio (S/N) method, where the LOD corresponds to the analyte concentration yielding an S/N of 3:1, while the LOQ was defined as the concentration achieving an S/N of 10:1.

### 2.5. Ecological Risk Assessment

#### 2.5.1. Calculation of Ecological Risk Assessment

The ecological risk assessment was conducted by calculating the risk quotient (*RQ*) for the compounds detected in surface water. The *RQ* for individual compounds was determined as the ratio of the measured environmental concentration (*MEC*) to the predicted no-effect concentration (*PNEC*), which is the maximum concentration at which no adverse effects on the ecosystem have been observed in the current study. The *RQsum* for mixtures was calculated by summing the individual *RQ* values [34]. The risk was categorized into four levels based on the ratio of the measured antibiotic concentration to the predicted no-effect concentration (*RQ*): negligible risk (*RQ* < 0.01), low risk (0.01 ≤ *RQ* < 0.1), moderate risk (0.1 ≤ *RQ* < 1), and high risk (*RQ* ≥ 1) [35].

(1) $$RQ=\frac{MEC}{PNEC}$$

The measured environmental concentration (*MEC*, ng/L) refers to the measured concentration of antibiotics, while the predicted no-effect concentration (*PNEC*, ng/L) is the maximum concentration at which no adverse effects on the ecosystem are expected. When multiple antibiotics are present in water, their combined toxicity is stronger than that of individual antibiotics [36]. The risk quotient (*RQ_i_*) for antibiotic i is calculated as follows: *RQ_i_* ≥ 1: high risk; 0.1 ≤ *RQ_i_* < 1: moderate risk; 0.01 ≤ *RQ_i_* < 0.1: low risk; *RQ_i_* < 0.01: negligible risk. The median effective concentration (*EC_50_*, ng/L) is the concentration at which 50% of the maximum effect is observed, and the assessment factor (*AF*) is used in the ecological risk evaluation. *PNEC* values were obtained from the literature (Table 3) or calculated as the ratio of *EC_50_* to *AF* [37].

The ecological risk of antibiotics in the groundwater and surface water of the Huixian Wetland was assessed using the combined risk quotient (*RQ_sum_*), calculated as follows:

(2) $$RQ_{sum}=\sum RQ$$

In aquatic environments, the ecological risk of antibiotics is commonly assessed using the risk quotient (*RQ*), calculated as follows:

(3) $$PNEC={EC}_{50}/AF$$

#### 2.5.2. Calculation of Health Risk Assessment

This study evaluated health risks using a risk quotient (*RQ*) model, with special consideration of risk variations across different age groups. The detailed calculation methods are expressed as follows:

(4) RQ=MEC/DWEL

(5) $$DWEL=\frac{ADI\times BW}{DWI\times AB\times FOE}$$

This study employed a risk quotient (*RQ*) model to assess health risks across different age groups, where *MEC* represents the measured antibiotic concentration (μg/L), *DWEL* denotes the drinking water equivalent level (μg/L), *ADI* corresponds to the acceptable daily intake (μg/(kg·d)), *BW* indicates the average body weight (kg), *DWI* refers to daily water intake (L/d), *AB* represents the gastrointestinal absorption rate (assumed as 1), and *FOE* signifies the exposure frequency (350 d/a, equivalent to 0.96). Parameters such as *BW* and *DWI* were derived from the Exposure Factors Handbook of Chinese Population [38], while antibiotic *ADI* values were obtained from the U.S. ECOTOX database or literature [39,40], with supplementary *BW* and *DWI* data sourced from the U.S. Environmental Protection Agency [41]. Risk levels were classified based on *RQ* thresholds: low risk (*RQ* < 0.1), moderate risk (0.1 ≤ *RQ* ≤ 1), and high risk (*RQ* > 1).

## 3. Results and Discussion

### 3.1. Antibiotic Concentrations in Wetland Surface Water

**Figure 2:**
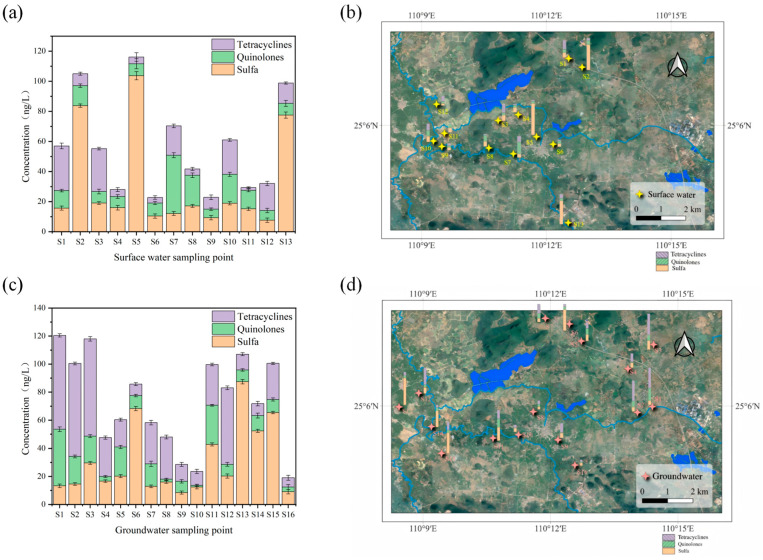
(**a**,**b**) Accumulated concentration of surface water at each sampling point. (**c**,**d**) Accumulated concentration of groundwater at each sampling point.

It can be seen from Figure 2 and Table 4 that among the 17 target antibiotics in the surface water of the Huixian Wetland, 16 were detected, including 8 sulfonamides (SAs), 4 quinolones (QNs), and 4 tetracyclines (TCs). Each sample is provided with three parallel samples. SAs were detected at all 13 surface water sites, with total concentrations ranging from 7.615 to 103.633 ng/L and an average of 31.226 ng/L. These included sulfadiazine (SDZ), sulfamethoxazole (SMZ), sulfamerazine (SMR), sulfamethazine (SMM), sulfapyridine (SMP), sulfachloropyridazine (SCP), sulfadoxine (SDM), and sulfamonomethoxine (SM2). Notably, SDZ, SMZ, SMM, SDM, and SCP were detected at a 100% frequency, with concentrations of 1.413–91.215 ng/L, 1.512–58.807 ng/L, 0.852–1.965 ng/L, 1.615–6.574 ng/L, and 0.618–0.898 ng/L, respectively. The average SA concentration was lower than that in the Caohai Wetland of Guizhou. In groundwater, all 16 sampling sites showed SA detections, with SDZ, SMZ, SMM, SDM, and SCP also exhibiting high detection frequencies (>90%). The detection rates of SAs in groundwater and surface water showed similarity, likely due to their high water solubility, strong environmental mobility, and weak adsorption properties. Surface water SAs accounted for 54.8% of the average antibiotic concentration, while groundwater SAs accounted for 41.8%. Compared to QNs and TCs, the higher usage of SAs in the Huixian Wetland surface area was evident. Among SAs, SDZ showed the highest detection concentration, with an average of 10.21 ng/L in surface water and 7.579 ng/L in groundwater. Higher concentrations were observed at surface water sites 5 and 13 and groundwater site 13, all located in agricultural areas, reaching 91.215, 15.658, and 46.656 ng/L, respectively. These averages were lower than those reported for Datzong Lake. The widespread use of sulfonamides in agriculture and aquaculture, along with their strong environmental mobility, contributes to their high detection rates and concentrations, particularly for SDZ and SDM [36].

Four QNs—norfloxacin (NOR), ciprofloxacin (CIP), enrofloxacin (ENR), and ofloxacin (OFL)—showed detection rates in surface water of 30.7%, 61.5%, 30.7%, and 76.9%, respectively, with total concentrations of 10.169–94.638 ng/L and an average of 41.828 ng/L. In groundwater, detection rates were 37.5%, 56.2%, 37.5%, and 93.4%, with total concentrations of 14.709–75.062 ng/L and an average of 51.905 ng/L. CIP and OFL exhibited particularly high detection rates (61.53% and 76.92% in surface water, 56.2% and 93.4% in groundwater) and concentrations: CIP ranged from 3.414–35.889 ng/L in surface water and 3.454–19.668 ng/L in groundwater; OFL ranged from 1.969–9.058 ng/L in surface water and 0.852–13.853 ng/L in groundwater. The highest OFL concentration in surface water was 9.058 ng/L at site 2. Compared to SAs, QNs showed lower detection rates and concentrations, likely due to their susceptibility to photodegradation and higher adsorption coefficients (K_d_), leading to their retention in sediment.

For TCs, doxycycline (DC), tetracycline (TC), chlortetracycline (CTC), and oxytetracycline (OTC) showed detection rates in surface water of 23%, 53.8%, 23%, and 53.8%, respectively. In groundwater, detection rates were 50%, 81.2%, 50%, and 87.5%, respectively. The higher detection rates in groundwater may be attributed to the photodegradability of TCs [42]. Since tetracyclines are easily adsorbed by sediments and particles and degrade readily, they are rarely detected in natural waters [38,39]. The relatively high detection rates of TCs in HuiXian Wetland suggest significant usage and discharge into the water body.

Compared to other domestic regions (Table 5), SA detection concentrations in Huixian Wetland surface water were much lower than in Baiyangdian and Taihu Lake, slightly lower than in Caohai Wetland, similar to Datzong Lake, and slightly higher than in the Huangpu River. QN concentrations were much lower than in Xining urban wetlands, slightly lower than in Caohai Wetland, Baiyangdian, Chaohu Lake, and Taihu Lake, similar to Datzong Lake, and slightly higher than in the Huangpu River. TC concentrations were much lower than in Taihu Lake, similar to Xining urban wetlands and Chaohu Lake, and slightly higher than in Datzong Lake. In groundwater comparisons (Table 6), SA concentrations were much lower than in Beijing groundwater, slightly lower than in Shanghai enterprise groundwater, similar to Shuanghe Cave and Dalian groundwater, and higher than in Jiangxi Jinjiang groundwater. QN concentrations were much lower than in Dalian groundwater, slightly lower than in Shuanghe Cave groundwater, similar to Beijing groundwater, and higher than in Jiangxi Jinjiang groundwater. TC concentrations were similar to Dalian groundwater but higher than in Shanghai enterprise groundwater, Shuanghe Cave groundwater, and Beijing groundwater.

### 3.2. Source Apportionment of Three Major Classes of Antibiotics in Surface Water and Groundwater of the Study Area

**Figure 3:**
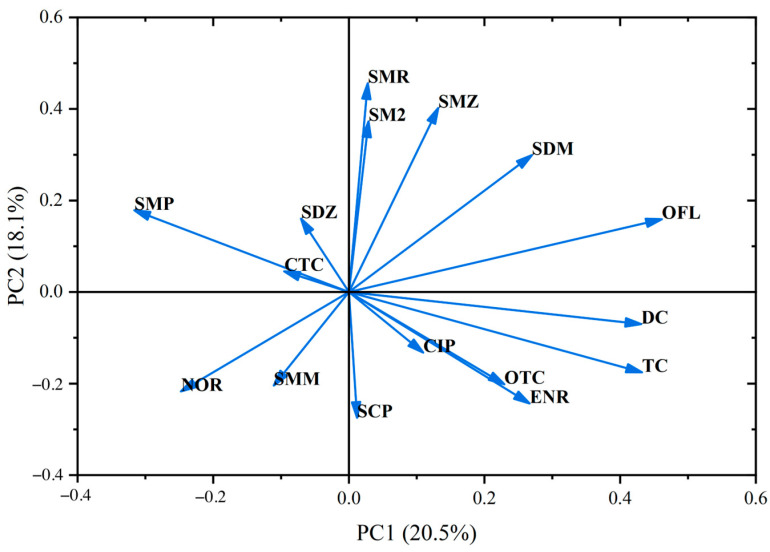
Principal component analysis diagram of antibiotics detected in the Huixian wetland of Guilin at sampling points.

To investigate the potential sources of the target antibiotics in surface water and groundwater, principal component analysis (PCA) was employed, yielding six principal components (PCs) as shown in Figure 3, collectively accounting for 75.776% of the variance. Specifically, PC1 explained 20.524% of the variance and had high loadings for SDM, SMP, NOR, ENR, OFL, TC, and DC; PC2 accounted for 18.061% and had high loadings for SM2, SDM, SMZ, SMR, SMM, and SCP; PC3 explained 14.208% and had high loadings for SDZ, SMM, SCP, NOR, CIP, OTC, and CTC; PC4, PC5, and PC6 explained 8.308%, 8.141%, and 6.534% of the variance, respectively, with high loadings for SMP, CIP, ENR, and OTC in PC4; SM2, SDZ, and OTC in PC5; and SMM and SCP in PC6.

Antibiotics with high loadings in PC1, such as SDM, SMP, NOR, ENR, OFL, TC, and DC, were detected in aquaculture areas, suggesting that the primary source of these antibiotics is aquaculture wastewater. For PC2, antibiotics with high loadings, including SM2, SDM, SMZ, SMR, SMM, and SCP, were predominantly detected in livestock farming areas, indicating that livestock wastewater is the main source. In PC3, antibiotics such as SDZ and SMM, which were detected at high concentrations in agricultural regions, are likely sourced from agricultural runoff.

The loadings of quinolones and tetracyclines in PC1 were higher than those of sulfonamides, while in PC2, the loadings of sulfonamides were higher than those of quinolones and tetracyclines. In PC3, the loadings of the three classes of antibiotics were comparable. This suggests that sulfonamide pollution primarily originates from livestock farming and agricultural runoff, whereas quinolone and tetracycline pollution mainly stems from aquaculture and agricultural runoff. These findings indicate that agricultural activities in the HuiXian Wetland are a significant contributor to the residual antibiotics in the water environment.

### 3.3. Ecological Risk Assessment

**Figure 4:**
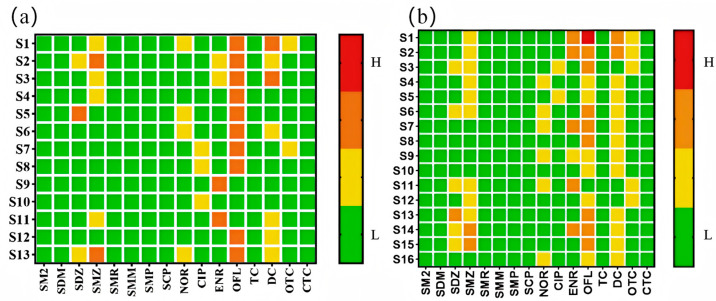
(**a**) Ecological risk heatmap of antibiotics in surface water of the Guilin Huixian Wetland. (**b**) Ecological risk heatmap of antibiotics in groundwater of the Guilin Huixian Wetland.

From Figure 4a,b, it is evident that none of the antibiotics in surface water posed a high ecological risk. However, five antibiotics—ofloxacin, sulfadiazine, sulfamethoxazole, enrofloxacin, and doxycycline—were at a moderate risk level, while the others exhibited low or negligible risks. In groundwater, one antibiotic, ofloxacin, presented a high risk, indicating acute toxicity to aquatic organisms, and it was detected in domestic sewage ditches. Four antibiotics (sulfadiazine, sulfamethoxazole, enrofloxacin, and doxycycline) were at a moderate risk, and the rest were at a low or negligible risk. The moderate-to-high risks of these five antibiotics in both surface and groundwater may be due to their high detection concentrations and extensive use in agriculture and aquaculture. Additionally, ofloxacin, enrofloxacin, and doxycycline were detected at higher levels near sites with frequent human activity.

**Figure 5:**
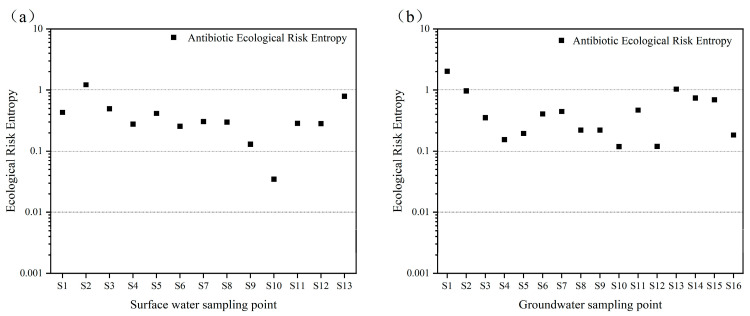
(**a**) Combined ecological risk entropy of antibiotics in surface water sampling sites. (**b**) Combined ecological risk entropy of antibiotics in groundwater sampling sites.

Antibiotic pollution in water is typically not from a single compound but from multiple antibiotics acting in concert. Studies have shown that the co-occurrence of multiple antibiotics or their interaction with other pollutants can lead to synergistic toxicity, amplifying health risks in aquatic environments. Therefore, assessing the ecological effects of individual antibiotics may underestimate their true environmental impact. To address this, a combined risk quotient (RQ_sum_) was used to evaluate the hazard at each sampling site. As shown in Figure 5, most sites were at a moderate-to-high risk, primarily due to the five aforementioned antibiotics. These results indicate that these five compounds contribute the most to the overall hazard to aquatic ecosystems and should be prioritized for control to protect water environments.

### 3.4. Health Risk Assessment

Based on the maximum detected concentrations of antibiotics in the Huixian Wetland and the acceptable daily intake (ADI) values obtained from the literature [55,56,57], a health risk assessment for different age groups was conducted (Table 7).

**Figure 6:**
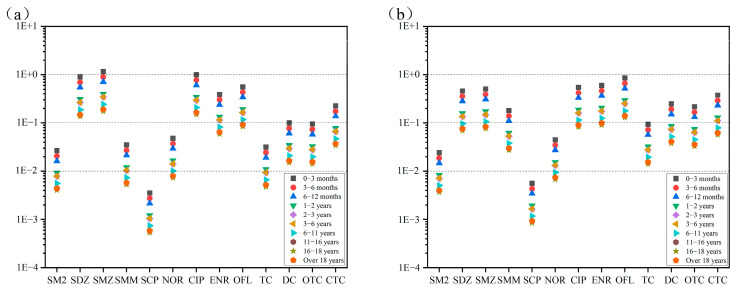
(**a**) Risk assessment of antibiotics in surface water on human health. (**b**) Risk assessment of antibiotics in groundwater on human health.

From Figure 6a,b, sulfadiazine, sulfamethoxazole, and ciprofloxacin in surface water posed a moderate health risk across all age groups, while ofloxacin, enrofloxacin, and chlortetracycline affected individuals under 16, 11, and 1 years old, respectively. Other antibiotics exhibited low or negligible risks. In groundwater, ofloxacin was the only antibiotic with a moderate risk across all ages, while sulfadiazine, sulfamethoxazole, sulfamethazine, ciprofloxacin, enrofloxacin, doxycycline, chlortetracycline, and oxytetracycline affected individuals under 11, 16, 1, 16, 16, 1, 1, and 6 years old, respectively. Overall, antibiotics in the Huixian Wetland primarily posed health risks to infants and adolescents, with less impact on adults.

## 4. Discussion

The findings of the present study on sulfonamides (SAs) in the Huixian karst wetland system align with and expand upon Qin research on antibiotic contamination in aquatic environments [32]. Compared to prior investigations, the detected SA concentrations in surface water (7.615–103.633 ng/L) and groundwater (mean: 7.579–7.087 ng/L for dominant SAs) were notably lower than those reported in intensive aquaculture or livestock regions, such as Baiyangdian Lake (505 μg/L for sulfadiazine) and Taihu Lake (4870 ng/L for sulfamethoxazole), but comparable to Datzong Lake and Huangpu River. This discrepancy likely stems from differences in local agricultural and aquaculture practices, as the Huixian Wetland’s pollution sources are dominated by decentralized livestock farming and limited aquaculture, contrasting industrialized regions. A spatial analysis revealed SA hotspots near agricultural zones (e.g., Site S12: 91.215 ng/L sulfadiazine), consistent with studies linking sulfonamide distribution to manure application and irrigation runoff. Temporal trends showed elevated concentrations during dry periods, attributed to reduced dilution effects, mirroring observations in Jianghan Plain groundwater. The ecological risk assessment identified sulfadiazine (RQ = 1.82–3.39 for algae) and sulfamethoxazole (RQ = 1.35–1.66) as moderate-to-high risks, corroborating risks reported in the Caohai Wetland and Gonghu Bay. However, the inclusion of quinolones and tetracyclines in the comparative analyses revealed that ofloxacin posed the highest cumulative risk (RQsum up to 4.7), underscoring the necessity of multi-class assessments to avoid underestimating mixed contamination effects. Health risks for infants (<1 year) from sulfamethazine (RQ = 1.39–1.82) aligned with groundwater studies in Beijing, while an expanded age-group analysis demonstrated adolescents’ susceptibility to fluoroquinolones (e.g., ofloxacin RQ = 0.33–0.88), a nuance often overlooked in singular SA-focused studies. A principal component analysis further clarified that 54.8% of SAs originated from agricultural runoff versus 41.8% from livestock, contrasting aquaculture-dominated sources in coastal wetlands. These findings collectively highlight that while Huixian’s SA levels are relatively low globally, their persistence in karst systems—coupled with mixture synergies and groundwater interconnectivity—necessitates targeted monitoring, particularly given the wetland’s role in the regional drinking water supply. The results emphasize context-dependent risk patterns, where moderate individual compound risks escalate to significant combined threats, urging integrated management strategies balancing agricultural practices and hydrological conservation in fragile karst ecosystems.

## 5. Conclusions

This study detected 16 antibiotics across three classes (sulfonamides, quinolones, and tetracyclines) in both surface water and groundwater of the Huixian Wetland. Sulfonamides dominated in surface water (average: 31.226 ng/L), followed by quinolones (41.828 ng/L) and tetracyclines, while groundwater showed a similar sulfonamide predominance (41.8% of total antibiotic concentration) but higher tetracycline detection rates. These findings align with prior studies in Chinese wetlands (e.g., Caohai, Baiyangdian) but highlight unique contamination patterns: lower sulfonamide levels than Caohai, comparable quinolone concentrations to Datzong Lake, and tetracycline detection rates exceeding those in natural waters due to localized agricultural and aquaculture inputs.

The principal component analysis traced antibiotic sources to aquaculture wastewater (PC1: 20.5% variance), livestock farming (PC2: 18.1%), and agricultural runoff (PC3: 14.2%), corroborating earlier work on antibiotic mobility and usage patterns. The ecological risk assessment identified ofloxacin (high risk in groundwater), sulfadiazine, sulfamethoxazole, enrofloxacin, and doxycycline as priority contaminants, consistent with their widespread use and environmental persistence reported in regions like Taihu Lake and Huangpu River. Health risks were moderate for infants/adolescents but negligible for adults, mirroring age-dependent vulnerability trends observed in other Chinese aquatic systems.

A key strength of this study lies in its dual assessment of surface and groundwater, providing a holistic view of antibiotic distribution rarely addressed in regional wetland studies. However, limitations include a restricted sampling scope (13 surface and 16 groundwater sites) and the exclusion of seasonal variation or sediment interactions, which may underestimate long-term risks. Future research should expand th spatiotemporal sampling, integrate a sediment-phase antibiotic analysis, and evaluate the synergistic effects of antibiotic mixtures to refine risk-management strategies. Prioritizing the monitoring of high-risk compounds like ofloxacin and investigating their degradation pathways in wetland ecosystems will enhance regulatory frameworks for aquatic conservation.

## Figures and Tables

**Table 1:** MS/MS MRM analysis parameters for antibiotics.

Analyte	CAS	Time (min)	Precursor Ion (m/z)	Product Ions (m/z)	Cone Voltage/V	Collision Energies/eV
SDZ	68-35-9	4.0	251	92.00/156.00	23	27/15
SMZ	723-46-6	7.5	254	92.00/156.00	27	26/16
SMR	127-79-7	5	265	92.00/156.00	24	28/15
SMM	57-68-1	5.9	279.1	92.00/186.00	30	28/16
SMP	80-35-3	5.8	281.1	92.00/156.00	26	30/17
SCP	80-32-0	7.2	285	92.00/156.00	22	28/15
SDM	2447-57-6	7.7	311	92.00/156.00	28	32/17
SM2	1220-83-3	7.7	281	92.00/156.00	28	31/22
TMP	738-70-5	5.9	291	123.00/230.00	35	27/25
NOR	70458-96-7	6.1	320.1	233.04/276.08	14	22/14
CIP	85721-33-1	6.4	332.1	231.01/288.08	2	30/16
ENR	93106-60-6	6.6	360.13	245.06/316.10	38	24/16
OFL	82419-36-1	5.8	362.11	261.07/318.08	4	24/18
TC	60-54-8	6.3	445.1	410.20/427.20	25	19/13
DC	564-25-0	8.2	445	410.20/428.20	26	24/19
OTC	79-57-2	6.5	461	426.20/443.20	23	20/13
CTC	57-62-5	8.1	479	444.20/462.20	27	23/19

**Table 2:** Retention times, linear Equations, correlation coefficients (R^2^), LODs, and LOQs for 17 antibiotics.

Antibiotic	Retention Time (min)	Linear Equation	Linear Range (μg/L)	LOD/(ng/L)	LOQ/(ng/L)
SDZ	3.71	y = 2013.71x − 6.71	2–400	0.03	0.11
SMZ	6.79	y = 2243.40x + 1250.97	2–400	0.04	0.11
SMR	4.53	y = 2268.55x + 84.59	2–400	0.01	0.04
SMM	5.41	y = 2.950.44x + 393.96	2–400	0.04	0.06
SMP	5.90	y = 3037.01x + 1028.63	2–400	0.12	0.36
SCP	6.45	y = 16,376.10x + 17,608.40	2–400	0.10	0.30
SDM	7.16	y = 4029.81x + 7179.02	2–400	0.01	0.03
SM2	7.00	y = 1495.33x − 193.07	2–400	0.16	0.42
TMP	5.03	y = 21,887.40x + 10,198.70	2–400	0.01	0.04
NOR	5.89	y = 1920.02x − 4365.11	2–400	1.02	3.25
CIP	6.13	y = 1982.54x − 3533.20	2–400	0.83	2.52
ENR	6.24	y = 33,711.90x + 12,404.50	2–400	0.01	0.04
OFL	5.54	y = 5011.41x − 2711.57	2–400	0.23	0.67
TC	5.86	y = 9073.67x − 7584.66	2–400	0.33	1.30
DC	5.98	y = 7057.79x − 11,026.3	2–400	0.63	2.49
OTC	6.16	y = 615.22x − 662.77	2–400	0.43	1.67
CTC	8.06	y = 412.68x − 241.75	2–400	0.25	0.89

**Table 3:** Toxicological data of different antibiotics for the most sensitive species.

Antibiotic	Test Species	Toxicity Data (mg·L^−1^)	Toxicity Type	Assessment Factor	PNEC (ng·L^−1^)
SDZ	*Plants*	*EC_50_* = 0.46	chronic	1000	460.0
SMM	*Alage*	*EC_50_* = 8.56	acute	1000	8.56 × 10^3^
SM2	*S. vacuolatus*	*EC_50_* = 19.52	acute	1000	1.952 × 10^4^
SDM	*Algae*	*EC_50_* = 401.92	acute	1000	4.02 × 10^5^
SMR	*Plants*	*EC_50_* = 0.68	acute	1000	680.0
SMP	*S. vacuolatus*	*EC_50_* = 3.82	acute	1000	3820
NOR	*V. fischeri*	*PNEC* = 1.038×10^−3^	chronic	100	103.8
CIP	*D. magna*	*EC_50_* = 1.30	acute	1000	1.3 × 10^3^
OFL	*P. subcapitata*	*NOEC* = 0.00113	chronic	100	11.3
DC	*P. subcapitata*	*NOEC* = 0.01	chronic	100	100.0
TC	*P. subcapitata*	*EC_50_* = 3.31	acute	1000	3.31 × 10^3^
OTC	*P. subcapitata*	*EC_50_* = 1.04	acute	1000	1.04 × 10^3^
CTC	*C. pyrenoidosa*	*EC_50_* = 9.31	acute	1000	9.31 × 10^3^

Note: EC_50_: median effective concentration; NOEC: no-observed-effect concentration; PNEC: predicted no-effect concentration.

**Table 4:** Antibiotic concentrations and detection rates in HuiXian Wetland waters.

Compound	Detection Rate (%)		Minimum (ng/L)		Maximum (ng/L)		Median (ng/L)		Mean (ng/L)		Standard Deviation	
	Surface Water	Groundwater	Surface Water	Groundwater	Surface Water	Groundwater	Surface Water	Groundwater	Surface Water	Groundwater	Surface Water	Groundwater
**SM2**	46.1	62.5	ND	ND	17.621	15.984	ND	4.521	6.358	4.496	4.856	4.902
**SDM**	46.1	37.5	ND	ND	4.721	4.642	ND	ND	4.516	1.669	2.345	2.226
**SDZ**	100	100	1.413	1.215	91.215	46.656	1.601	2.124	10.210	7.579	24.705	11.613
**SMZ**	100	100	1.512	1.612	58.807	25.648	2.645	3.452	9.242	7.087	17.086	7.761
**SMR**	100	100	0.852	0.693	1.965	1.696	1.158	0.925	1.201	1.027	0.337	0.306
**SMM**	100	100	1.615	1.895	8.885	45.895	2.894	2.885	3.562	6.788	2.055	11.702
**SMP**	92.3	87.5	ND	ND	1.499	1.495	1.385	1.275	1.338	1.144	0.390	0.463
**SCP**	100	93.4	0.618	ND	0.898	1.425	0.725	0.825	0.757	0.816	0.088	0.272
**TMP**	0	0	ND	ND	ND	ND	ND	ND	ND	ND	0.000	0.000
**NOR**	30.7	37.5	ND	ND	2.796	2.587	ND	ND	2.542	0.919	1.224	1.228
**CIP**	61.5	56.2	ND	ND	35.889	19.668	3.652	4.125	11.830	4.691	10.697	5.959
**ENR**	30.7	37.5	ND	ND	12.286	18.835	ND	ND	6.711	3.875	3.734	6.429
**OFL**	76.9	93.4	ND	ND	9.058	13.853	2.836	2.812	3.566	3.491	2.432	3.639
**TC**	23.1	50	ND	ND	4.801	14.253	ND	3.858	4.423	3.407	1.951	4.316
**DC**	53.8	81.2	ND	ND	15.25	37.858	1.617	2.882	6.600	6.345	5.136	9.471
**OTC**	23.1	50	ND	ND	14.417	32.985	ND	3.612	10.428	6.983	5.086	9.994
**CTC**	53.8	87.5	ND	ND	22.878	38.241	3.012	13.295	10.829	12.929	7.833	11.988

Note: ND = not detected.

**Table 5:** Antibiotic concentrations in surface water of selected domestic regions (ng/L).

Study Area	Datzong Lake	Caohai Wetland	Baiyangdian	Chaohu Lake	Huangpu River	Taihu Lake	Xining Urban Wetland
**SDZ**	11.65–100.21	ND–130.2	0.86–505	ND–45. 6	ND–24.02	——	——
**SMZ**	ND–50.09	0.590–172	ND–940	ND–137.9	1.26–25.07	ND–114.7	——
**SMM**	ND–0.16	ND–41.4	ND–16.1	ND–4.7	ND–7.382	ND–654	——
**NOR**	ND–0.2	1.37–116	ND–156	ND–34.8	ND	ND–6.5	1.9–520.86
**CIP**	ND–5.02	1.09–11.4	ND–63	ND–13.6	ND	ND–43.6	ND–24.5
**ENR**	ND–38.14	0.85–19.4	ND–4.42	ND–82.7	ND	——	2.46–27.06
**OFL**	ND–40.17	ND–14.6	0.38–32.6	1.2–182.7	ND	ND–82.8	ND–1220.86
**TC**	ND–4.04	——	——	ND–9.8	——	ND–87.9	ND–16.16
**DC**	——	——	——	ND–42.3	——	——	ND–2.22
**OTC**	ND–3.66.	——	——	ND–4.9	——	ND–72.8	ND–30.72
**CTC**	ND–10.44	——	——	ND–4.4	——	ND–142.5	ND–14.88
**Reference**	[43]	[44]	[45]	[46]	[47]	[48]	[49]

Note: ND = not detected. —— = not tested.

**Table 6:** Antibiotic concentrations in groundwater of selected domestic regions (ng/L).

Study Area	Shanghai Enterprise	Shuanghe Cave	Dalian	Jiangxi Jinjiang	Beijing
**SDZ**	ND–93.6	ND	ND–52	ND	ND–96.8
**SMZ**	ND–17.4	ND–30.7	ND–42	18.1–29.7	ND–8.4
**SMM**	ND–23.4	ND–0.23	Nd–8	ND–0.26	ND–236
**NOR**	——	ND–15.53	ND–78	ND	ND–0.9
**CIP**	——	10.13–34.33	ND–2	ND	ND–9.2
**ENR**	——	ND–13.28	ND–17	ND–1.91	ND–39.4
**OFL**	——	10.18–34.68	ND–45	ND–1.81	ND
**TC**	——	ND	ND–2	ND	ND
**DC**	——	ND	ND–53	ND–1.56	ND
**OTC**	——	ND	ND–24	ND–2.65	ND–3.2
**CTC**	——	ND–15.97	ND–16	ND–4.18	ND
**Reference**	[50]	[51]	[52]	[53]	[54]

Note: ND = not detected. —— = not tested.

**Table 7:** ADI values for different antibiotics.

Antibiotic	ADI (μg·kg^−1^·d^−1^)	Antibiotic	ADI (μg·kg^−1^·d^−1^)
SM2	130	CIP	7.1
SDZ	20	ENR	6.2
SMZ	10	TC	30
SMM	50	DC	30
SCP	50	OTC	30
NOR	11.4	CTC	20
OFL	3.2		

## Data Availability

The original contributions presented in the study are included in the article, and further inquiries can be directed to the corresponding authors.
